# Celebrating the struggle against homophobia, transphobia and biphobia as central to ending HIV transmission by 2030

**DOI:** 10.1002/jia2.25532

**Published:** 2020-05-14

**Authors:** Tonia C Poteat, Stefan Baral

**Affiliations:** ^1^ Department of Social Medicne University of North Carolina School of Medicine Chapel Hill NC USA; ^2^ Department of Epidemiology Johns Hopkins School of Public Health Baltimore MD USA

**Keywords:** homophobia, transphobia, biphobia, HIV, stigma, violence

Sixteen years ago, Louis Georges Tin, advocate for Black and lesbian, gay, bisexual, transgender and intersex (LGBTI) rights, launched an appeal for universal recognition of May 17 as the International Day Against Homophobia to honour the World Health Organization’s decision to remove homosexuality from their list of mental disorders in 1990 [1]. Over time, the movement has grown and made explicit its relevance to all people with diverse sexual orientations, gender identities, gender expression and sex characteristics. May 17 is now commemorated as the International Day Against Homophobia, Transphobia and Biphobia (IDAHOT) in more than 130 countries, including 37 where same‐sex acts are illegal [2].

This year’s theme “Breaking the Silence” calls for an end to the stigma and violence that drive shame, increase HIV vulnerability, and hinder access to and uptake of HIV prevention and care services [3]. As the world grapples with the COVID‐19 pandemic, the novel coronavirus is highlighting existing disparities with disproportionate deaths among populations made most vulnerable by structural violence and discrimination, including LGBTI people. At the same time, some state actors are misusing emergency powers enacted to fight the pandemic to target LGBTI communities with structural and physical violence [4]. This year’s IDAHOT theme calls on us to speak out against this violence, not only because freedom from violence is a universal human right but also because doing so is essential to the goal of ending HIV as a pandemic by 2030 [5, 6].

In 2020, the status of protective and punitive laws affecting the sexual, reproductive and human rights of LGBTI communities around the world is dynamic [7]. Some countries have decriminalized same‐sex practices, whereas others have reinforced criminalization; some countries have increased restrictions on organizations serving LGBTI groups; others have increased constitutional rights, whereas others have further restricted them; and finally, some countries have enacted specific protections for LGBTI people from discrimination and from sexual orientation and gender identity change efforts or “conversion therapy,” whereas others have backtracked those same protections. While more resource‐constrained settings tend to have more restrictive legal contexts secondary to colonialism and ongoing neocolonialism, notably, there is no clear division by geography, income level or development index that separates countries where LGBTI rights are advancing and those where there have been setbacks. Moreover, there is no connection between rights contexts for LGBTI individuals and whether the country in which they are a citizen are signatories of the UN Declaration of Human Rights, which was intended to define fundamental human rights to be universally protected [8]. To clarify international principles specific to sexual orientation and gender identity, the Yogyakarta Principles were originally developed in 2006 [9, 10]. The goal of these Principles was to further articulate legal standards to protect the health and wellbeing of LGBTI communities around the world. The Yogyakarta Principles were updated in 2017 with what was called the Yogyakarta Principles plus 10 (YP+10), which focused on principles and obligations specific to sexual orientation, gender identity, gender expression and sex characteristics [11]. YP+10 reinforced that all LGBTI people have a right to simply live free of criminalization for who they are and who they love. Importantly, YP+10 reinforced the links between these rights and health consistent with the WHO definition as more than the absence of disease but attaining physical and mental wellbeing.

There are data supporting the harmful effects of stigmas against LGBTI people at every step of the cascade limiting achieving coverage of evidence‐based HIV prevention strategies, testing approaches, linkage to treatment for those living with HIV and sustained viral suppression. Indeed, many of the innovations in HIV were developed to specifically mitigate the harmful effects of these intersecting stigmas including implementation strategies such as HIV self‐testing, app‐based surveillance and service delivery strategies, and outreach‐based linkage and treatment services. And while many of those innovations have been successful in improving outcomes, they do not change the underlying constructs which reinforce individual HIV risks. Often, we see the language of non‐heteronormative practices such as anal sex as being “risky.” However, consensual anal sex is a healthy sexual practice. “Risk” is introduced in the context of serodifferent sexual partners having condomless anal sex where one is viraemic. And the introduction of that risk is a failure at many levels including limited capacity in health centres to provide sex‐positive education for clients about anal sex, limited LGBTI‐focused community‐based organizations to support outreach, limited availability of condom and appropriate lubricant choices, and of course intersecting stigmas in health centres challenging testing and linkage to treatment for those living with HIV. The international community tends to focus stigma mitigation efforts on the last piece of this—stigma in the health centre [12]. Indeed, to truly overcome disproportionate HIV‐related risks among LGBTI communities around the world means a fundamental shift in how we conceptualize sex and love from risk mitigation to celebration.

Combatting stigma, discrimination and violence against sexual and gender minorities requires action at multiple levels and across many sectors. Laws and policies that penalize same‐sex relationships and consensual sexual practices, prohibit marriage between consenting adults, and criminalize gender identity and expression should be repealed in keeping with universal human rights standards. Sexual orientation and gender identity change efforts have been shown to cause significant harm, especially for LGBTI youth, and should not be sanctioned by any accrediting body or government [13, 14]. Anti‐stigma interventions using participatory theatre, professional training and other modalities have been effective in reducing interpersonal stigma and healthcare stigma that underpin experiences of LGBTI violence [15, 16, 17]. Ensuring that healthcare workers are trained to provide welcoming and competent care for LGBT people is key to engaging key populations effectively in HIV prevention and care and to promoting an effective human rights‐based response to the COVID‐19 pandemic. Importantly, researchers and implementers must continue to reach and engage LGBTI communities as partners in efforts to develop, test and implement acceptable, effective stigma‐reducing, anti‐violence interventions needed to truly end new HIV infections by 2030.

## Competing interests

The authors declare no conflicts of interest.

## Authors’ contributions

TP and SB each wrote sections of the initial draft, reviewed all revisions and approved the final version of the manuscript.
